# Evaluation of biochemical profile of Chronic Kidney Disease of uncertain etiology in Sri Lanka

**DOI:** 10.1371/journal.pone.0232522

**Published:** 2020-05-04

**Authors:** Buddhi N. T. W. Fernando, Thilini S. H. Sudeshika, Thilini W. Hettiarachchi, Zeid Badurdeen, Thilak D. J. Abeysekara, Hemalika T. K. Abeysundara, Sakunthala Jayasinghe, Shirani Ranasighe, Nishantha Nanayakkara

**Affiliations:** 1 Department of Medical Laboratory Science, Faculty of Allied Health Sciences, University of Ruhuna, Matara, Sri Lanka; 2 Department of Pharmacy, Faculty of Allied Health Sciences, University of Peradeniya, Peradeniya, Sri Lanka; 3 Faculty of Medicine, Centre for Education, Research and Training on Kidney Diseases (CERTKiD), University of Peradeniya, Peradeniya, Sri Lanka; 4 Department of Statistics and Computer Science, Faculty of Science, University of Peradeniya, Peradeniya, Sri Lanka; 5 Department of Pathology, Faculty of Medicine, University of Peradeniya, Peradeniya, Sri Lanka; 6 Department of Biochemistry, Faculty of Medicine, University of Peradeniya, Peradeniya, Sri Lanka; 7 Transplant and Dialysis Unit, Teaching Hospital, Kandy, Sri Lanka; University of KwaZulu-Natal, SOUTH AFRICA

## Abstract

Chronic Kidney Disease of uncertain etiology (CKDu) is an endemic, disease that mostly affects young agricultural workers in the rural dry zone of Sri Lanka. This study was designed to identify specific biochemical manifestations of CKDu cases. All (119) non-dialysis definite CKDu patients in Girandurukotte and Wilgamuwa were selected. Blood and urine samples were collected and measured biochemical parameters. All analyses were performed in IBM SPSS statistics version 23 (IBM Corp, USA). The median blood pressure was normal though nearly half of the patients (45.4%) who were in the advanced stages (Stage 3b, 4 and 5) of CKDu. Patients without a history of hypertension before the diagnosis of CKDu (100%) and minimal proteinuria (26%) are similar to the previous findings. Patients without a history of diabetes before the CKDu diagnosis had high percentages of diabetes (15.7%) and pre-diabetes (59.8%) and hence indicated the possibility of uremia induced impaired glucose intolerance in the rural areas of the country. There were 62.2% patients who had low vitamin D and only a minority had evidence of bone mineral diseases. Out of liver disease markers serum glutamic pyruvic transaminases (SGPT), serum glutamic oxaloacetic transaminases (SGOT), gamma-glutamyl transferase (GGT), and Lactic acid degydrogenase (LDH) had an inverse correlation with the advancement of the disease indicating subclinical liver disease. Osmolality in serum and urine showed a discrepancy despite > 50% of CKDu patients had increased their serum osmolality. The current study supports most of the previously described manifestations of CKDu. Moreover, some specific patterns have been identified which need to be validated in a larger group.

## Introduction

Chronic Kidney Disease (CKD) is predominantly due to diabetes or hypertension, associated with unhealthy lifestyle in the growing elderly population not only in industrialized countries but also in some developing countries [1]. Recent epidemiological studies revealed that CKD is more prevalent in Asian countries than in developed Western countries [2,3]. Nagata et al (2010) have described that the prevalence of CKD would increase over time as a result of the accumulation of risk factors such as hypertension, glucose intolerance, obesity and hypercholesterolaemia, probably due to the westernization of the lifestyle even in these Asian countries [4]. In addition to these traditional causes, an emergence of CKD of uncertain etiology (CKDu) has been reported in poor rural farming communities of tropical countries like El Salvador, Egypt, India, Nicaragua and Sri Lanka since 1990s [5].

**Fig 1:**
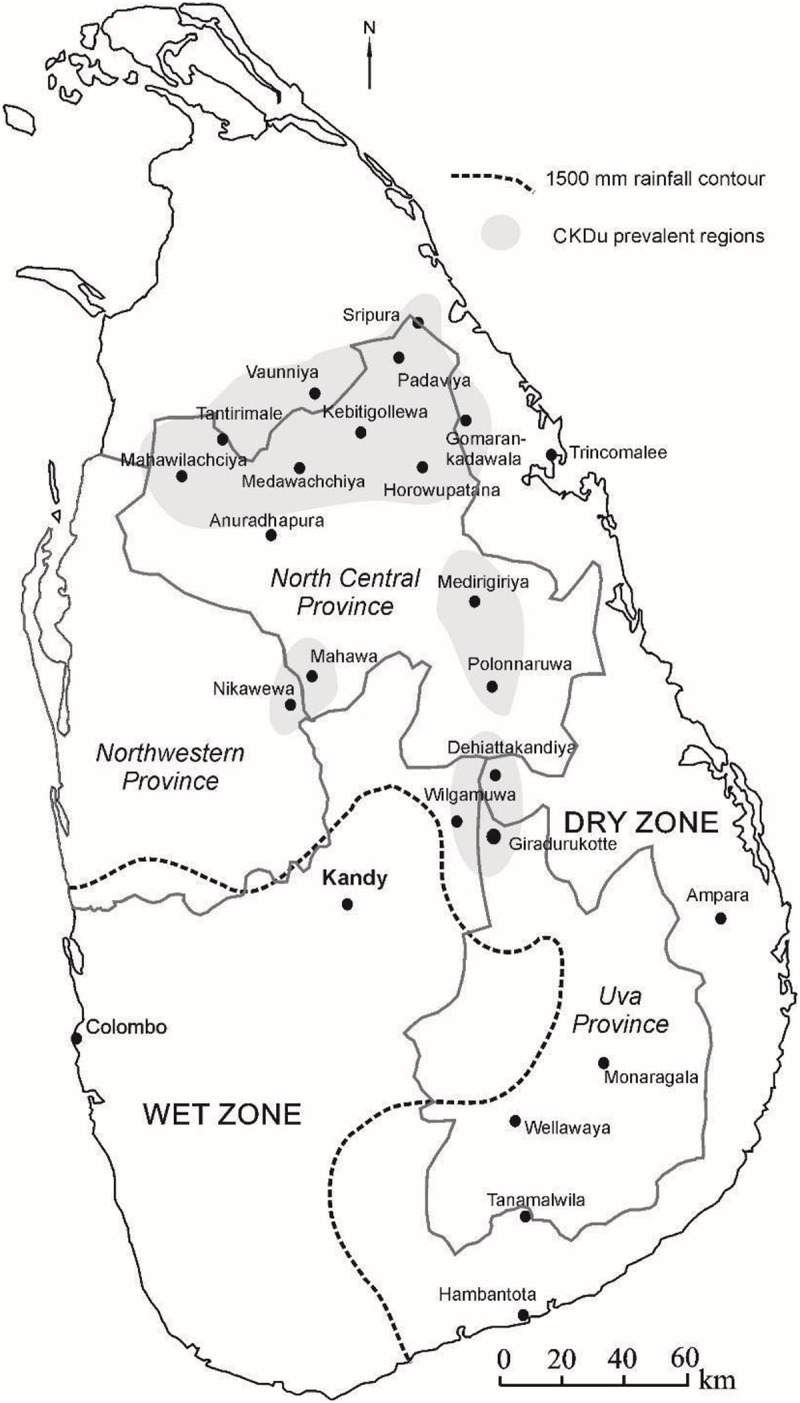


Many synonyms like, chronic kidney disease of unknown or uncertain origin, chronic kidney disease of unknown etiology, CKD of nontraditional causes and chronic interstitial nephritis in agricultural communities have been used in medical literature to define this disease [1]. In Sri Lanka, a remarkable increase in renal diseases has been reported during the time period between 1990 and 2007, which later named as CKDu. CKDu is endemic among farming communities in the dry zone of Sri Lanka [6–8] (Fig 1).

For the histological evidence in CKDu of chronic interstitial nephritis, as the causative agent, an environmental toxin was most probably suggested [9]. Vervaet et al (2019) have described that exposure to potentially toxic agrochemicals through ingestion of contaminated food, drinking water via contaminated wells, inhalation and direct contact is a cause for the chronic interstitial nephritis in agricultural communities in the world including Sri Lanka [9]. Other evidences are epidemics of environmental or occupational exposure related nephropathies like Aristolochic acid nephropathy (Balken nephropathy), Pb Nephropathy (Australia) and Cd nephropathy (Itai-itai disease in Japan) have been described in the medical history of different parts of the world. All these diseases typically cause tubular interstitial disease similar to CKDu with specific manifestation of the toxin. Malignancies in Balken nephropathy, painful bone disease in Cd nephropathy and neuropathy and macrocytic anemia in Pb nephropathy are some examples of extra renal manifestations of causative agents [10–13].

Pathophysiology of CKD is complex but mostly due to long-term derangement of metabolism leading to Bone Mineral Diseases, anemia, increased Cardiovascular Disease (CVD) risk, glucose intolerance, acidosis, hyperuricemia and electrolyte abnormalities [14]. These manifestations could be superimposed by population specific factors obesity, over nutrition, diabetes, dyslipidemia in rich countries, malnutrition in poor countries and endemic factors in CKDu [7].

This study was designed to identify the unique characteristics of CKDu in Sri Lanka, by analyzing key biochemical parameters of non-dialysis definite CKDu patients.

## Materials and methods

### Study design, setting and clinical data collection

All definite CKDu cases (132: male 108, female 24) in two renal clinics were enrolled after informed written consent during the period of June 2015 to February 2017 part (S1 Table). Case definition was applied according to the S1 Table which was developed by Teaching Hospital, Kandy (S1 Table). The ethical clearance was granted by the Ethical Review Committee of the Faculty of Medicine, University of Peradeniya (2016/EC/28). A detailed history was obtained using a structured questionnaire including demographic data (age, gender, body mass index [BMI], hypertension and diabetes mellitus after diagnosis of CKDu. Blood samples (10ml) were collected from peripheral veins into K-EDTA tubes, plain tubes and Na-citrated tubes. Serum was separated immediately after clotting by centrifugation at 3000rpm for 10 minutes. Spot urine samples were collected from all the recruited cases into empty, sterile, polypropylene urine containers. All the serum and urine samples collected were processed for routine biochemical parameters on the same day of the sample collection. Laboratory parameters were measured in a clinical laboratory, the Teaching Hospital, Kandy. Most of the biochemical parameters were measured using Indiko plus Analyzer (Thermo Scientific^™^, Finnland) and serum electrolyte were measured using Electrolyte machine (BioCare BIOLYTE 2000, Taiwan). Serum and urine osmolality were measured using Osmometer (OsmoTECH^R^ 3320, Norwood). Full Blood counts were analyzed using haematology analyzer (Mindray BC 5300, China). Hormones were analyzed using Chemiluminescence Immunoassay Analyzer (MAGLUMI 600, Chennai). Proteinuria was detected by using 3% sulphosalicillic acid. Estimated glomerular filtration rate (eGFR) was calculated using the Chronic Kidney Disease-Epidemiology Collaboration (CKD-EPI) equation [15].

#### Statistical analysis

All analyses were performed in IBM SPSS statistics version 23. Continuous variables were reported as means (mean ± SD) whereas categorical variables were expressed as the number and the proportions. Further Spearman Rank correlation and Pearson Rank correlation were calculated to determine the correlations. For the mean comparison with other studies, two sample T test was done. In all analysis, P < 0.05 was considered as significant.

## Results

**Fig 2:**
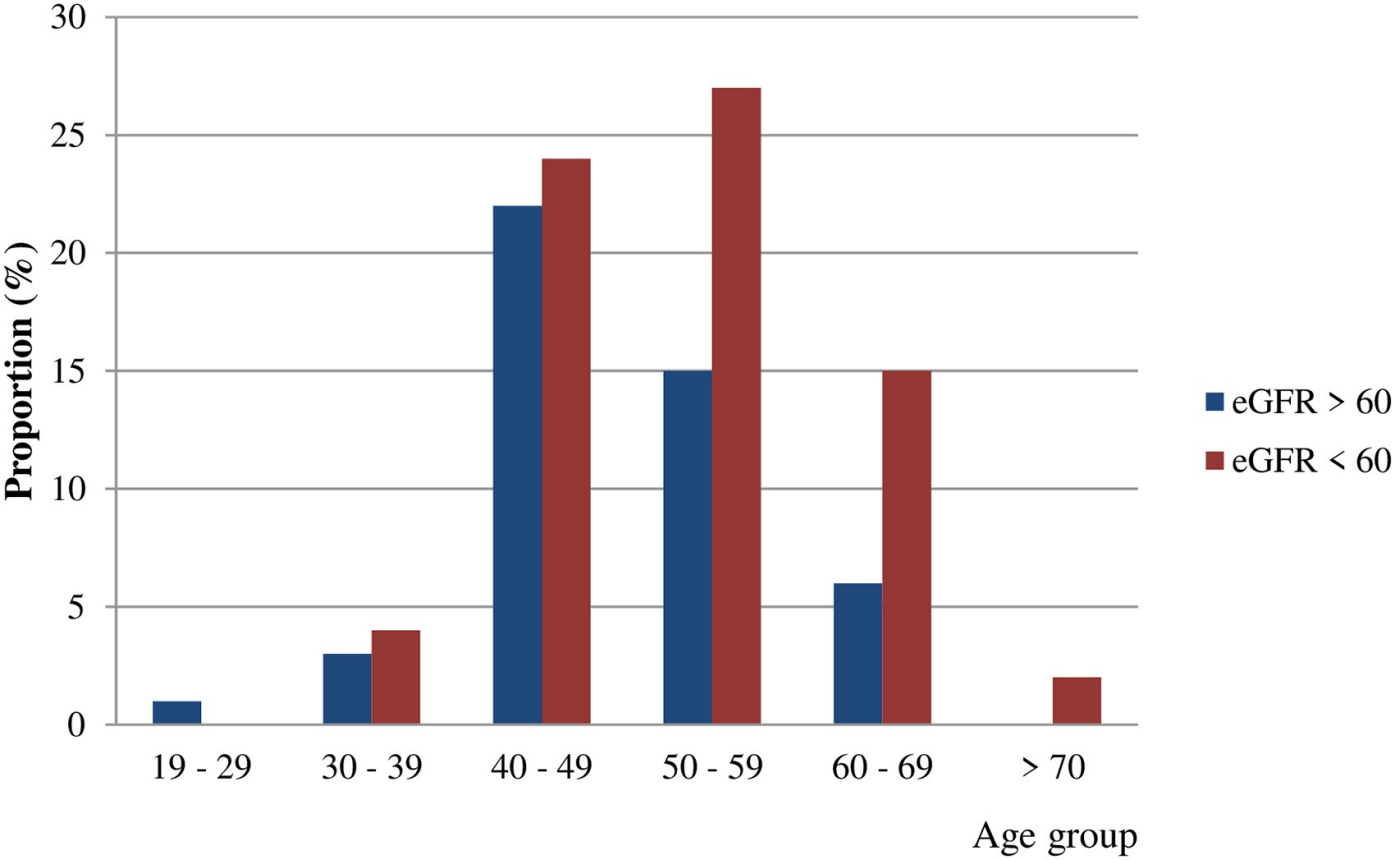
High and low estimated glomerular filtration rate (eGFR) for age in decades among CKDu patients, n = 119. eGFR calculated by eGFR-EPI formula [15].

Out of 132 recruited patients, only 119 (97 males, 22 females) had participated in all the laboratory investigations. Demographic data, life style and health characteristics of the non-dialysis patients with definite CKDu are shown in Table 1. Seventy-four percent of patients were between, 40 to 60 years (Fig 2). Median BMI of CKDu patients was within the normal range. Higher BMI (> 25 kg/m^2^) was observed in 20.2% of CKDu patients while it was 14% for lower BMI (< 18 kg/m^2^). The median blood pressure was normal and only 13.4% were on more than one antihypertensive despite the fact that nearly half of the patients (45.4%) were in the advanced stages of the disease (stage 3b, 4 and 5). Out of total subjects, 74% were farmers. Fifty-one percent of patients had family history of CKD. Among the life style habits, 78% of patients had the habit of chewing betel which was higher than smoking and alcohol consumption. Thirty percent of the patients had developed hypertension and 6% of the patients had developed Diabetes after the diagnosis of CKDu. Interestingly, the number of Diabetes were 15.7% according to HbA1C results (HbA1C > 6.5) indicating undetected disease among this population. Alarmingly 59.8% were in pre-diabetes state (HbA1C 5.5 to 6.5) with higher chance of developing Diabetes in the future.

**Table 1:** Demographic and clinical characteristics of the study subjects, n = 119.

Variable	Median (IQR) or cases (Percentage %)
Age, years	51 (11)
Sex distribution: Males	97 (81.5%)
Weight, Kg	58 (12.62)
Height, m	1.63 (1.07)
BMI, Kg/m^2^	22 (4.9)
Systolic BP, mmHg	120 (10)^a^
Diastolic BP, mmHg	80 (10)
Occupation as a farmer	88 (74%)
Family history of CKD	61 (51%)
Smoking (males only)	48 (40%)
Chewing betel	93 (78%)
Alcohol (males only)	50 (42%)
Medical History: Hypertension	36 (30%)
Diabetes mellitus	7 (6%)
Malaria History	45 (38%)

Values are expressed as numbers with proportion of populations for each characteristic (%), or as mean ± s.d., as appropriate.

^a^ Four patients had increased systolic blood pressure

SD, standard deviation; BMI, body mass index; BP, blood pressure; CKD, chronic kidney disease

Median levels and clinical significance of biochemical laboratory findings in serum of CKDu patients are presented in Table 2. Clinical significance of all the parameters were detected using the reference ranges established in the kit inserts of each test reagent. They are presented in the Table 2. The median level for all the blood investigations are within the reference interval except for the raised serum creatinine, uric acid, amylase, alkaline phosphatase and decreased vitamin D (Table 2).

**Table 2:** Biochemical laboratory findings in serum of CKDu patients in Girandurukotte and Wilgamuwa (n = 119).

No	Parameter	Median (IQR)	Range	Normal N (%)	Low N (%)	High N (%)	Reference Range
1	Sodium, mmol/L	142 (8.45)	128–156	64 (53.8)	28 (23.5)	27 (22.7)	136–145
2	Potassium, mmol/L	4.5 (0.8)	3.1–6.0	98 (82.4)	5 (4.2)	16 (13.4)	3.5–5.1
3	Calcium, mg/dL	9.1 (0.65)	7.4–10.5	98 (82.4)	19 (16)	2 (1.6)	8.6–10.3
4	Phosphorous, mmol/L	1.04 (0.24)	0.48–2.15	96 (80.7)	17 (14.3)	6 (5.0)	0.87–1.45
5	Creatinine, μmol/L	136.1 (119.7)	39–810	40 (33.6)	NA	79 (66.4)	M < 113
							F < 96
6	Urea, mg/dL	27.2 (17.4)	9.6–75.9	95 (79.8)	4 (3.4)	20 (16.8)	13–43
7	Uric Acid, mg/dL	6.6 (2.2)	1.8–11.2	78 (65.5)	NA	41 (34.5)	M- 3.5–7.2
							F- 2.6–6.0
8	Total Protein, g/L	72 (5)	61–88	116 (97.5)	2 (1.7)	1 (0.8)	64–83
9	Albumin, g/L	43.6 (3.2)	38–52	118 (99)	NA	1 (0.8)	35–52
10	Amylase, U/L	142.18 (74.1)	55–383	24 (20.2)	NA	95 (79.8)	< 100
11	LDH, U/L	210.4 (53.4)	149–325	70 (58.8)	NA	49 (41.2)	135–225
12	Bicarbonate, mmol/L	26.0 (4.7)	15–33	83 (69.7)	20 (16.8)	16 (13.5)	22–29
13	GGT, U/L	25.5 (17.5)	12–236	102 (85.7)	NA	17 (14.3)	M < 55
							F < 38
14	SGPT, U/L	22 (19.3)	8–171	97 (81.5)	NA	22 (18.5)	M < 45
							F < 34
15	SGOT, U/L	24.5 (10.8)	11–110	98 (82.4)	NA	21 (17.6)	M < 35
							F < 31
16	SGOT/SGPT	1.07 (0.5)	0.50–2.5	52 (44)	NA	67 (56)^a^	< 1.00
17	ALP, U/L	125.2 (98.9)	33–2229	44 (37)	1 (0.8)	74 (62.2)	35–105
18	Vitamin D, ng/mL	26.6 (19.6)	13–100	44 (37)	74 (62.2)	1 (0.8)	30–100
19	PTH, pg/mL	55.1 (28.4)	7.9–144	103 (86.6)	NA	16 (13.4)	6–80
20	CRP, mg/L	4.5 (0.5)	3.0–20.2	108 (91.5)	NA	10 (8.5)	≤ 6.0
21	HbA1C, % (n = 102)	5.6 (0.5)	4.5–15^b^	25 (24.5%)	NA	16 (15.7)H	<5.5 Normal
						61 (59.8)I	5.5–6.5Impaired(I)
							> 6.5 High (H)
22	T.Cholesterol, mmol/L	4.9 (1.3)	2.2–7.6	69(58.0)	36 (30.2)	14(11.8%)	<5.2 desirable
							5.2–6.2Borderline high
							>6.2 High
23	Magnesium, mmol/L	0.71 (0.15)	0.44–10.85	79 (66.4)	35 (29.4)	5 (4.2)	0.66–1.07
24	TSH, μIU/mL	1.40 (1.89)	0.01–23.3	96 (80.7)	13 (10.9)	10 (8.4)	0.3–4.5
25	Ferritin,ng/mL (n = 114)	64.11 (94.4)	4.35–439.31	98 (86)	11 (9.6)	5 (4.4)	M 25–350
							F 13–232
26	Calcitonin, pg/mL	13.1 ± 19	10–211	114 (95.8)	NA	5 (4.2)	< 18
27	Hemoglobin, g/dL	12.2 (2)	8.6 ± 15.3	33 (27.7)	86 (72.3)	NA	M < 13
							F < 12
28	White cell count, cells/mm^3^	6990 (2655)	3180–14150	109 (91.6)	NA	10 (8.4)	4000–10000
29	S.Osmolality, mOsm/Kg (n = 118)	295 (16.75)	230–413	56 (47.4)	4 (3.4)	58 (49.2)	275–295
30	U.Osmolality,mOsm/Kg (n = 118)	371.5 (281.7)	102–750	74 (62.7)	42 (35.6)	2 (01.7)	300–900

^a^ Six patients (5%) had SGOT/SGPT ratio as > 2.0

^b^ Seven cases were diagnosed with diabetes after the diagnosis of CKDu

Abbreviations: SD, standard deviation; LDH, lactate dehydrogenase; GGT, gamma glutamil transferase; SGPT, serum glutamate-pyruvate transaminase; SGOT, serum glutamate-oxaloacetate transaminase; ALP, alkaline phosphatase; PTH, parathyroid hormone; CRP, c reactive protein; TSH, thyroid stimulating hormone; M, male; F, female; S, serum; U, urine; NA, not available

**Fig 3:**
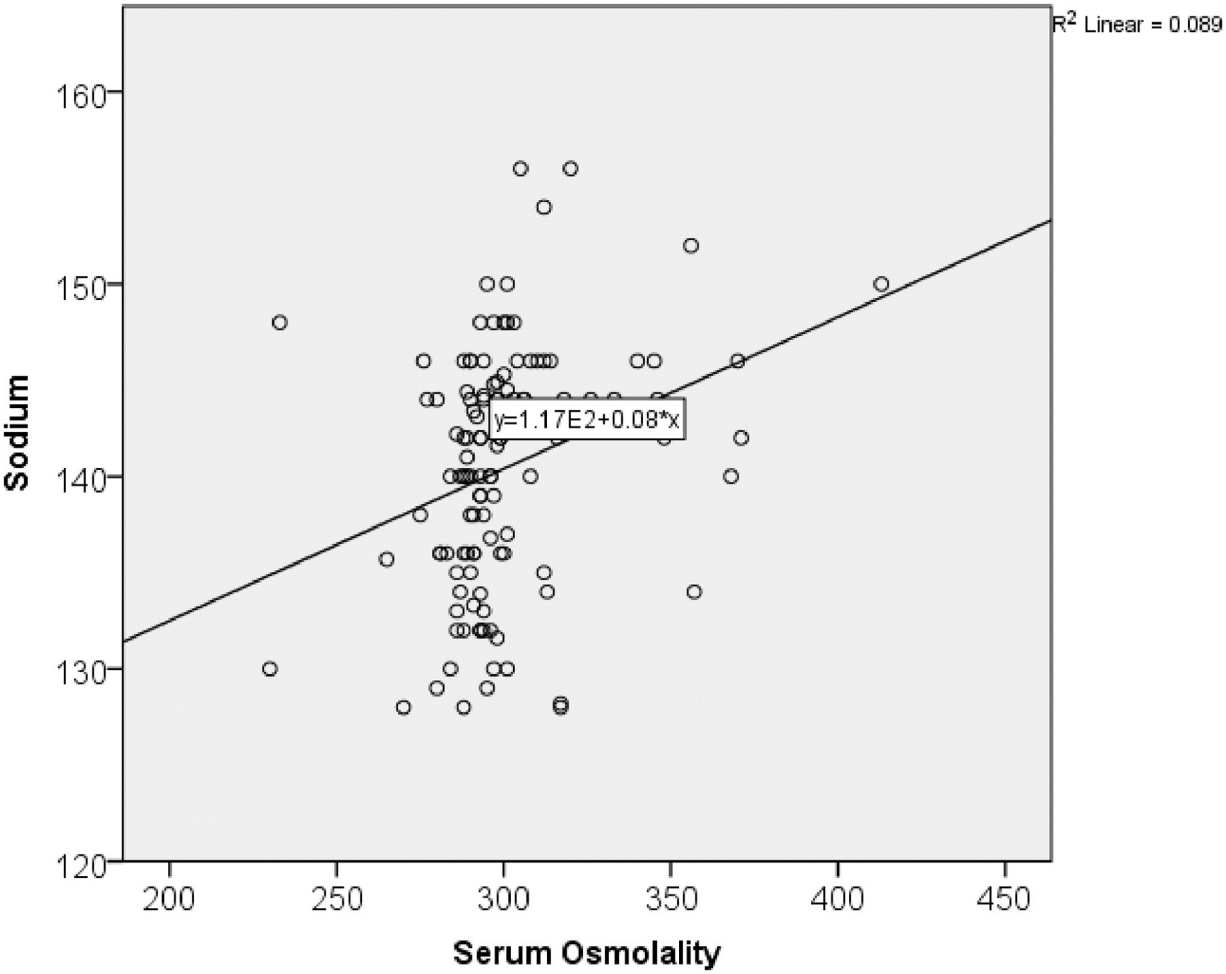
Correlation of serum sodium with serum osmolality. Serum sodium was significantly positively correlated with the serum osmolality.

Only some CKDu patients had hyperuricemia (34.5%), hypophosphatemia (5%), hypocalcemia (13.4%) and acidosis (16.8%). Alkaline phosphatase (62.2%) and amylase (79.8%) were increased in a majority. An inflammatory marker, C-reactive protein (< 6mg/L) was normal in all participants. Sixty two (62%) of CKDu patients had low levels of vitamin D (< 30 ng/mL) while other hormones (TSH, PTH, Ferritin, Calcitonin) remained normal in > 80%. More than 50% of CKDu patients had increased serum osmolality. Out of the study population, 72.3% had hemoglobin levels below the reference interval. Table 3 shows the correlation of some renal markers. According to those results, urine osmolality did not significantly nor positively correlate with the serum osmolality. But sodium was significantly positively correlated with the serum osmolality (Fig 3). BMI was significantly positively correlated with the HbA1C and the cholesterol. According to the HbA1C results, prevalence of Diabetes and pre-diabetes were 15.7% and 59.8% respectively. Serum Mg was not correlated significantly with serum creatinine and eGFR. Serum LDH was significantly positively correlated with the SGPT, SGOT and GGT and negatively correlated with ALP and serum creatinine. Serum amylase significantly and positively correlated with serum creatinine. SGPT and SGOT significantly and negatively correlated with serum creatinine while GGT also showed negative correlation with serum creatinine which was not significant. Vitamin D positively correlated with the serum creatinine but was not significant.

**Table 3:** Correlations of important markers of CKDu patients.

Parameter	Correlate with;	r	p
S.Osmolality	U.Osmolality	0.117	0.206
	Na	0.363**	<0.001
BMI	S.Cholesterol	0.191	0.042
	S.Albumin	-0.130	0.167
	HbA1C	0.270**	0.007
Mg	Creatinine	-0.112	0.226
	eGFR	0.120	0.193
LDH	CRP	0.060	0.522
	Hb	0.033	0.721
	SGPT	0.249**	0.006
	SGOT	0.386**	<0.001
	GGT	0.220*	0.016
	ALP	-0.096	0.300
	U.Acid	-0.002	0.983
	Creatinine	-0.191*	0.038
	eGFR	0.161	0.080
SGPT	Creatinine	-0.368	<0.001
SGOT	Creatinine	-0.389	<0.001
GGT	Creatinine	-0.126	0.172
Amylase	Creatinine	0.383**	<0.001
	eGFR	-0.380	<0.001
Vitamin D	Creatinine	0.179	0.051

**. Correlation is significant at the 0.01 level (2-tailed)

* Correlation is significant at the 0.05 level (2-tailed).

Abbreviations: BMI, body mass index; HbA1C, glycosylated hemoglobin; Mg, Magnesium; eGFR, estimated glomerular filtration rate; LDH, lactate dehydrogenase; CRP, c reactive protein; SGPT, serum glutamate-pyruvate transaminase; SGOT, serum glutamate-oxaloacetate transaminase; GGT, gamma glutamil transferase; ALP, alkaline phosphatase; U.Acid, uric acid

In the urine analysis, urine Sulfosalicylic acid test showed abnormal urine protein (+, ++, +++ or > +++) in 31 patients (26%). Only Four patients (3.4%) had abnormal urine sugar. In sediment analysis, 20 patients (16.8%) had > 4 leukocytes per high-power field. Only 11 patients (9.2%) had > 4 erythrocytes per high power field. One patient (0.8%) had few granular casts and six patients (5%) had calcium oxalate crystals in their urine sediments.

Table 4 shows the comparison of the findings with the previous studies. According to this Table, similar pattern can be observed in the current study in BMI, blood pressure, sodium, potassium, calcium, serum albumin and white cell count when compared to the previous studies. When compared with the study done by Nanayakkara et al (2014), a significant difference was found in the BMI, diastolic blood pressure & HbA1C [16]. When compared with the study done by Aturaliya et al (2011), a significant difference was found in the BMI, K, uric acid, phosphorous and Hb while others were not significant [7]. According to the results of the study done by Wijkstrom et al (2018), most of the parameters of the current study are compatible with that study while increased average level of magnesium and decreased level of uric acid were seen in the current study [17].

**Table 4:** Comparison of parameters of CKDu patients with previous studies.

Parameter	Nanayakkara et al(2014) (n = 311) Mean (SD) [16]	Wijkstrom et al (2018) (n = 11) Mean (SD) [17]	Aturaliya et al (2011) (n = 109) Mean (SD) [7]	Current Study (n = 119) Mean (SD)	Nanayakkara et al vs Current study (P value)	Aturaliya et al vs current study (P value)
BMI, (kg/m^2^)	21.1 (3.4)	20 (3)	21 (2.7)	22.1 (3.6)	0.01*	0.009*
Systolic BP, mmHg	124.0 (18.9)	122 (20)	127 (45)	124 (13)	1	0.5
Diastolic BP, mmHg	71.0 (12.6)	78 (9)	81 (23)	78 (7)	0.000*	0.193
HbA1C, %	5.5 (0.6)	NA	NA	6.0 (1.0)	0.000*	NA
Na, mmol/L	NA	140 (4)	139.14 (4.66)	140.4 (6)	NA	0.07
K, mmol/L	NA	4.3 (1)	4.13 (0.62)	4.5 (0.6)	NA	0.000*
Mg, mmol/L	NA	0.70 (0.16)	NA	0.93 (1.25)	NA	NA
Uric Acid, mg/dl	NA	7.06 (1.6)	5.84 (0.75)	6.6 (1.7)	NA	0.000*
Ca, mg/dL	NA	9.0 (1.6)	9.02 (1.54)	9.1 (0.5)	NA	0.6
Albumin, g/L	NA	38 (7)	NA	44 (2)	NA	NA
Phosphorous, mmol/L	NA	1.0 (0.2)	1.53 (0.42)	1.06 (0.24)	NA	0.000*
Hb, g/dL	NA	12.4 (1.9)	12.76 (2.98)	12.1 (1.5)	NA	0.039*
WBC, 10^6^/L	NA	7350 (1350)	NA	7279 (1871)	NA	NA

SD, standard deviation; BMI, body mass index; BP, blood pressure; HbA1C, glycosylated hemoglobin; Hb, hemoglobin; WBC, white blood cells; NA, not available;

*, Significant difference *p* < 0.05

## Discussion

CKDu is an environmental nephropathy reported from Central America, India, Taiwan and Sri Lanka causing significant morbidity and mortality. Natural history, clinical and biochemical manifestations are important in predicting complications and determining treatment strategies of a disease. There was limited literature on biochemical profile in CKDu. Most findings were normal or compatible with CKD and can be easily overlooked in a superficial evaluation. This is the first study which describes the biochemical profile of non-dialysis patients with the diagnosis of definite CKDu, in Sri Lanka.

In comparison to other forms of CKD, there are several described characteristics features in CKDu. Young or middle-aged male farmers were mostly affected by CKDu [18]. The peak affected age of CKD correlated with life expectancy of the country. In Iraq with a life expectancy of 74.9 years [19], peak age of CKD was 60 years [20]. In western countries, with a life expectancy more than 80 years [19], peak incidence is found at the age of 75 years [21]. The peak age of present study group was 51 years which is unusual for a country with an average life expectancy of more than seventy years. Alarmingly, more than 50% of cases in the present study had family history indicating the genetic tendency of CKDu [18, 22].

Dyslipidemia, hypoalbuminemia and impaired glucose tolerance were already described adverse metabolic phenomena in CKD. Obesity is a major health concern worldwide and identified as a risk factor for CKD and CVD [23]. According to a prevalence study done by Katulanda et al (2018) [24], 77.4% of Sri Lankan adults had some form of dyslipidaemia. Interestingly only 11% of CKDu patients had high serum cholesterol and only 22.7% were on lipid lowering medications. Even though the prevalence of obesity was 20% and lower than CKD (31.6%) [25], it was higher than previously described in CKDu (0%) [7, 17], and lower than Mesoamericam Nephropathy in Costa-Rica (26.4%) [26]. BMI is significantly positively correlated with the HbA1C and the serum cholesterol. Katulanda et al (2008) described the community prevalence of diabetes and pre diabetes was 8.7% and 11% in rural Sri Lanka [27]. Prevalence of 15.7% and 59.8% for Diabetes and pre-diabetes respectively were alarmingly high in CKDu indicating the effects uremia induced impaired glucose intolerance and changing life style in the rural areas of the country. Positive correlation of BMI with HbA1C and serum cholesterol further supports this possibility. Findings warrant prompt intervention to prevent major consequences in affected communities.

Along with other chronic interstitial diseases minimal proteinuria, normoalbuminemia and normal blood pressure have been already identified as features of CKDu [7]. Current study showed essentially normal serum albumin and only 26% had dipstick proteinuria more than one plus which was inexplicable for CKD. Hypoalbuminemia and albuminuria were common in CKD (78.4%) [28], but unusual in CKDu. In a recent study both parameters were identified as discriminating features of CKDu or interstitial diseases from CKD even in non-endemic areas [29].

Tubular proteinuria and various tubular manifestations were common in interstitial nephropathies [7]. Nanayakkara et al (2012) indicated a tubular marker, Alpha 1 microglobulin was elevated in more than 55% of CKDu cases and sensitive marker for early diagnosis and screening [30] and some studies have been described other interstitial nephropathies [31,32]. There were several studies investigating the effectiveness of tubular markers, including KIM1 and NGAL for screening and diagnosis of CKDu [30, 33–35]. Even though tubular manifestations like hypokalaemia, hyperuricaemia, renal tubular acidosis, have been described in CKDu [36]. In contrast our results did not support the existence of such manifestations in CKDu. More than 50% had increased osmolality and a significant positive correlation with serum sodium. In physiological status serum osmolality positively correlates with the urine osmolality. Conversely such correlation was not found in this group indicating a tubular non-responsiveness despite dehydration.

Exact health effects of Mg in general population or CKD have not been properly investigated. Mountokalakis (1990) described the limited excretion of Magnesium in CKD could leads to toxic levels causing adverse effects [37]. Even though the cause is not obvious lower Mg in the later stages of CKDu, was conflicting with the observations in CKD [20]. It is interesting to study the effect of low calcium intake, low dietary Mg intake, tubular dysfunction, effects of drugs and role of genetic predisposition like HFi beta mutation in hypomagnesemia.

Though the phosphate retention and hyperphosphatemia were extremely common in patients with End Stage Renal Disease and CKD [38], only minority of our group had bone mineral disease abnormalities. Findings are partially explained by vegetable based Sri Lankan diet with low phosphate. Most of the CKDu patients in the present study suffered by vitamin D deficiency which is compatible with a study done by Wesseling-Perry et al (2012) that showed a decreased average level of vitamin D in CKD patients [39]. The patients with CKDu who were in late stages had better level of vitamin D probably due to vitamin D supplementation.

Serum amylase levels are increased in patients with renal insufficiency due to decreased excretion of the enzyme [40]. The elevation of amylase in the present study supports the possibility by showing significant positive correlation with serum creatinine. Almost 50% of the population had high LDH and it was negatively corelate with creatinine. Five isoenzymes of LDH have been discovered and could be due to pathological changes in liver, bone, hematological system, muscles or infections and cancers [41]. Along with LDH, SGPT, SGOT, and GGT had a similar association with lowering levels in the advancement of the disease. In a study done on CKD patients in India, serum transaminases showed a similar lowering with the severity of CKD increases [42]. Fabrizi et al (2001) also reported that serum ALT levels were reduced concomitantly with the progression of renal dysfunction [43]. SGOT/SGPT, more than one is associated with various pathological forms of hepatitis [44]. CKDu is possibly a toxin mediated disease and raised the possibility of sub clinical hepatitis as an extra renal manifestation caused by the same agent, especially in early disease.

The study was conducted in CKDu definite cases which support most of the previously described manifestations of CKDu. This study was conducted in biopsy confirmed patients and thereby limiting the number of cases. Secondly, the study was designed to identify typical biochemical profile, specific manifestations and differences from already described manifestation for CKD. Interestingly, some specific patterns have been identified which need to be validated in a larger group. Evidence of super added metabolic syndrome on CKDu, higher levels of serum Mg at earlier stages, role of vitamin D deficiency on BMD and possible subclinical hepatitis were interesting manifestations and need further evaluation.

The main strength of the study is the detailed description of groups of patients with biopsy definite CKDu patients with biochemical data from serum and urine. All the tests were analyzed with quality controls at the renal unit laboratory, Teaching Hospital, Kandy. There are some limitations with the study. The major factor among the study’s limitations is its small sample size. There is a lack of establish a causal relationships between the outcome and exposure because the nature of the study design. The cross-sectional design precludes causal relationships and thus further prospective studies should be undertaken. This is a preliminary study and these specific patterns have been identified in the current study need to be validated in a larger group.

## Supporting information

S1 Table Case definition of Chronic Kidney Disease of unknown etiology (CKDu) Sri Lanka. (PDF)
