# Correction: Evaluation of biochemical profile of Chronic Kidney Disease of uncertain etiology in Sri Lanka

**DOI:** 10.1371/journal.pone.0238279

**Published:** 2020-08-20

**Authors:** Buddhi N. T. W. Fernando, Thilini Sudeshika, Thilini W. Hettiarachchi, Zeid Badurdeen, Thilak D. J. Abeysekara, Hemalika T. K. Abeysundara, Sakunthala Jayasinghe, Shirani Ranasighe, Nishantha Nanayakkara

The second author's name is spelled incorrectly. The correct name is: Thilini Sudeshika. The correct citation is: Fernando BNTW, Sudeshika T, Hettiarachchi TW, Badurdeen Z, Abeysekara TDJ, Abeysundara HTK, et al. (2020) Evaluation of biochemical profile of Chronic Kidney Disease of uncertain etiology in Sri Lanka. PLoS ONE 15(5): e0232522. https://doi.org/10.1371/journal.pone.0232522
