# Supplementary material for: Evaluation of biochemical profile of Chronic Kidney Disease of uncertain etiology in Sri Lanka
Source: PLoS One. 2020 May 4;15(5):e0232522. doi: 10.1371/journal.pone.0232522 (PMC7197770; doi:10.1371/journal.pone.0232522)
Supplement: S1 Table — (PDF) [file pone.0232522.s001.pdf]

Supplementary Table 01: Case definition of CKDu in SriLanka, 2018 developed by Teaching hospital, Kandy

Case definition of Chronic Kidney Disease of unknown etiology (CKDu) Sri Lanka 2018 Update
**Suspected CKDu**
**Essential criteria**
eGFR < 60 mL/min using CKD EPI equation: One time measurement using standardized methods for creatinine measurement<br>OR albuminuria ≥ 30 mg/g creatinine<br>OR proteinuria ≥ 150mg/g creatinine
**Exclusion criteria - to identify suspect CKDu among those satisfying above criteria**
Urine protein: creatinine ratio > 3000mg/g creatinine<br>OR urine albumin: creatinine ratio >300mg/g creatinine
Diabetes based on self-report of diagnosis OR being on treatment OR capillary random plasma glucose ≥ 200 mg/dL
Hypertension based on treatment with more than two drugs OR untreated blood pressure of more than 160/100 mmHg (preferably using electronic BP apparatus, sitting position, at least two readings one minute apart)
Acute kidney injury that required dialysis in the past based on the history or documented evidence
Age >70
**Probable CKDu**
**Essential criteria**
**Exclusion criteria to identify probable CKDu among those satisfying above criteria**
Diabetes based on the presence of any of the standard criteria for diagnosis (fasting plasma glucose ≥126mg/dl, 2-hour plasma glucose ≥200mg/dl on oral glucose tolerance test, HbA1c ≥6.5%)

Clinical OR laboratory OR ultrasound evidence of other known causes of CKD such as<br>• polycystic kidney disease<br>• congenital malformations<br>• autoimmune diseases<br>• glomerular diseases
Ultrasound evidence of<br>• unequal kidney sizes with a discrepancy of >1.5cm<br>• obstructive nephropathy<br>• kidney stones of any of the following features<br>o an obstructive stone<br>o a non-obstructive single stone of >10mm in size<br>o a non-obstructive multiple stones of more >5mm in size in either or both kidneys
**Confirmed CKDu**
**Confirmed with histopathology consistent with CKDu**
All the above mentioned criteria for probable CKDu<br>AND (in addition)<br>Histopathological features consistent with CKDu on biopsy
**Confirmed clinically in the absence of histopathology**
All the above mentioned criteria for probable CKDu<br>AND (in addition)<br>Renal biopsy not possible
